# Association between obesity indices and cardiovascular risk factors in late adolescence in the Seychelles

**DOI:** 10.1186/1471-2431-12-176

**Published:** 2012-11-07

**Authors:** Pascal Bovet, Tiphaine Arlabosse, Bharathi Viswanathan, Gary Myers

**Affiliations:** 1 Institute of Social and Preventive Medicine (IUMSP), Lausanne University Hospital, Lausanne, Switzerland; 2 Section of Noncommunicable Diseases, Ministry of Health, Victoria, Republic of Seychelles; 3 Department of Child Neurology, University of Rochester Medical Center, Rochester, NY, USA

**Keywords:** Obesity, Body mass index (BMI), Cardiovascular risk factors, Screening, Youth, Adolescents, Young adults, Africa

## Abstract

**Background:**

The ability of different obesity indices to predict cardiovascular risk is still debated in youth and few data are available in sub Saharan Africa. We compared the associations between several indices of obesity and cardiovascular risk factors (CVRFs) in late adolescence in the Seychelles.

**Methods:**

We measured body mass index (BMI), waist circumference, waist/hip ratio (WHiR), waist/height ratio (WHtR) and percent fat mass (by bioimpedance) and 6 CVRFs (blood pressure, LDL-cholesterol, HDL-cholesterol, triglycerides, fasting blood glucose and uric acid) in 423 youths aged 19–20 years from the general population.

**Results:**

The prevalence of overweight/obesity and several CVRFs was high, with substantial sex differences. Except for glucose in males and LDL-cholesterol in females, all obesity indices were associated with CVRFs. BMI consistently predicted CVRFs at least as well as the other indices. Linear regression on BMI had standardized regression coefficients of 0.25-0.36 for most CVRFs (p<0.01) and ROC analysis had an AUC between 60%-75% for most CVRFs. BMI also predicted well various combinations of CVRFs: 36% of male and 16% of female lean subjects (BMI <P50) had ≥2 CVRFs compared to 74% of male and 46% of female overweight subjects (BMI >P90).

**Conclusion:**

There was an elevated prevalence of obesity and of several CVRFs in youths in Seychelles. BMI predicted single or combined CVRFs at least as well as other simple obesity indices.

## Background

Obesity in youth has important short term and long term impact on health [1], including increased cardiovascular mortality and morbidity in middle age [2-4]. The detrimental impact of obesity on the incidence of subsequent cardiovascular disease (CVD) is partly mediated by increased levels of cardiovascular risk factors (CVRF), in particular hypertension, dyslipidemia and diabetes [4].

The comparative value of different indices of obesity for the prediction of cardiovascular outcomes is still debated. This question is differently framed whether the emphasis is to predict: i) the actual adiposity status, which relies on the comparison of indices of obesity with objective measurements of general/central fat, ii) subsequent cardiovascular outcomes, which relies on cohort studies [2,4], or iii) the presence of concomitant CVRFs [5,6], which can be assessed with cross-sectional studies. In this paper, the focus is on the performance of indices of obesity to predict the presence of concomitant CVRFs in young adults.

Several studies and reviews have examined the performance of different obesity indices to predict the concomitant presence of abnormal levels of CVRF in youths including high blood pressure, dyslipidemia and diabetes [5]. BMI, which is a simple and widely used metric, has been criticized as an inadequate measure of obesity because it does not distinguish well between lean mass and fat mass. Other advocated indices include waist circumference [7,8], waist-to-hip ratio (WHiR) [9], waist-to-height ratio (WHtR) [10,11] or percent body fat based on bioimpedance [12]. Mixed results have been reported, but several studies and reviews have concluded that BMI performed as well as other obesity indices for the identification of an adverse cardiometabolic risk profile in youths [13-15].

The performance of anthropometric indices may vary according to various factors, including race, geographical area, and population [16]. However, few studies have examined the performance of anthropometric indices for the identification of adverse cardiovascular risk profiles in sub Saharan Africa populations [17-19], and none among representative samples of youths. A challenge for such studies is the need to collect biological samples (e.g. blood lipids) from healthy young individuals. It is important to collect information on the associations between obesity indices and CVRFs in African populations to guide recommendations for surveillance and clinical practice in the region, particularly in the context of the rapidly rising prevalence of obesity worldwide, including in the Seychelles [20].

In this study, we examined the associations between several obesity indices and the main CVRFs in a population-based sample of late adolescents of the Seychelles aged 19–20 years. Seychelles is a middle-income country in the African region. The study also provides useful information on the prevalence of the major CVRFs in youths in this region.

## Methods and Procedures

### Subjects

The Republic of Seychelles is a small island state in the Indian Ocean located east of Kenya. The majority of the population is of African descent.

Between February 1989 and February 1990, a cohort of 779 children was enrolled at the age of 6 months as part of the Seychelles Child Development Study. This ongoing cohort study was undertaken to examine the association between exposure to methyl mercury from fish consumption and the children’s neurological outcomes. The study methodology was previously described [21]. Study participants were evaluated in 2008 and 2009 at age 19–20 years. Cardiovascular outcomes were evaluated including neurovegetative and cardiometabolic ones. Informed written consent for participation was obtained from all participants. The protocol was approved by the institutional review boards of the University of Rochester Medical Center and the Republic of Seychelles.

### Evaluation

Anthropometric data were measured without shoes and in light garments. Weight was measured using a precision electronic scale (Seca 870, Hamburg, Germany) and height was measured with a fixed stadiometer (Seca 208). Body mass index (BMI) was calculated as weight (kg) divided by height (meters) squared. Waist circumference was measured at mid distance between the floating rib and the iliac crest at the end of normal expiration with a standard tape measure. Hip circumference was measured to the nearest 0.1 cm at the point of maximum extension of the buttocks. Body fat percent was assessed using a bioimpedance analyzer (Omron HBF-306C). All anthropometric indices were measured once. Blood pressure (BP) was based on the average of three readings taken on the left arm with a validated oscillometric automated device (Omron M5, Omron Healthcare Europe BV, Hoofddorp, The Netherlands) using an appropriately sized cuff, after a 5 minute rest. Three senior nurses with experience in conducting epidemiological research performed the measurements. When comparing mean values of the measurements in participants between the observers’ groups, adjusting for sex and age, there was no difference between for height, weight, waist, WHiR, BMI, fat percent and systolic/diastolic BP, but a statistically significant difference was found for hip and WHtR. This suggests lower validity of measurements involving hip. A structured questionnaire was self-administered to assess alcohol consumption and smoking status. Tobacco use and alcohol use were assessed with the questions: “During the past 30 days, on how many days did you [smoke at least on cigarette] [have at least one drink]?” with answers to chose among categories: 0 day; 1–2 days; 3–5 days; 6–9 days; 10–19 days; 20–29 days; and every day. Smoking was defined for participants reporting to smoke at least one cigarette every day. Physical activity was assessed using the Global Physical Activity Questionnaire (GPAQ) [22].

### Biological sampling

A venous blood sample was collected between 7 and 9 am after an overnight fast. Glucose, total cholesterol, HDL-cholesterol, triglycerides and uric acid were measured at the central laboratory of the main hospital in the Seychelles (Victoria Hospital), using standard enzymatic methods (Konelab T series reagents, Thermo Scientific) with a Thermo Konelab 30 automatic analyzer (Konelab Corp., Espoo, Finland). LDL-cholesterol was calculated with the Friedewald formula.

### Statistical analyses

We conducted analyses separately in males and females because of substantially different distributions of several variables. Since the aim of the study is the prediction of the presence of concomitant CVRFs according to various indices of obesity, main analyses are not adjusted for other covariates. We first examined the association between the obesity indices and CVRFs along continuous scales using linear regression. We display the standardized regression coefficients, which express a difference in the “outcome” variable corresponding to a difference of 1 standard deviation in the “exposure” variable. This permits direct comparison of the regression coefficients between different obesity indexes and across the different CVRFs. We then estimated the prevalence of elevated levels of CVRFs according to increasing categories of obesity indices. We categorized all obesity indices in five categories (i.e. percentiles <50, 50–79, 80–89 and 90–100). We performed ROC (Roc Operating Characteristics) analysis [23] to evaluate the performance of the indices of obesity: the area under the curve (AUC) statistic represents the probability of correctly identifying subjects with or without the outcome of interest (i.e. CVRFs). An AUC of 0.5 means that prediction is not better than chance and 1.0 represents perfect discrimination. We defined dichotomized categories of CVRFs based on recent guidelines for subjects aged 18–21 [24]. Because only 4% of subjects had triglycerides ≥1.3 mmol/l, we used a lower cutoff (≥1.0 mmol/l), which resulted in a prevalence of 12%. There is no widely used cutoff for serum uric acid in youth and we defined elevated levels as above the sex-specific 80^th^ percentile. Finally, we examined the prevalence of combinations of the 6 considered CVRFs across categories of the obesity indices. Analyses were performed using Stata 11.2. We used two-sided tests and considered statistical significance as P <0.05.

## Results

The distribution of the obesity indices and the CVRFs is shown in Table 1. Males were taller and heavier than females but BMI was higher in females than in males (27% of females and 13% of males had BMI ≥25 kg/m^2^). Waist circumference was similar in both sexes but the proportion with elevated waist circumference was higher in females than in males. While the waist/hip ratio (WHiR) was larger in males than in males, the waist/height (WHtR) ratio and the percentage of body fat were larger in females than in males. There were marked differences in the distribution of CVRFs according to sex. Males smoked more, consumed more alcohol, and had higher levels of physical activity than females. Systolic BP but not diastolic BP was markedly higher in males than in females. Males had higher LDL-cholesterol and lower HDL-cholesterol levels than females, but mean triglyceride levels were similar in both sexes. Mean levels of blood glucose and uric acid were higher in males than in females.

**Table 1:** Distribution of obesity indices and cardiovascular risk factors

	**Males (n=195)**		**Females (n=228)**		***P***
***Anthropometric variables***					
Height (cm)	175.0	6.8	162.5	6.7	0.000
Weight (kg)	66.2	12.2	59.8	14.6	0.000
Body mass index (kg/m^2^)	21.6	3.6	22.7	3.3	0.016
≥ 25	13.3		26.7		0.000
≥ 30	4.6		10.5		0.000
Waist (cm)	75.9	8.6	75.7	11.2	ns
≥ 94 (M); ≥80 (F)	5.6		28.5		0.000
≥102 (M); ≥88 (F)	2.1		14.9		0.000
Waist/height ratio	43.3	3.5	46.6	4.6	0.000
≥0.50	9.7		26.7		0.000
Waist/hip ratio	0.83	0.07	0.82	0.09	0.017
Fat percent	16.8	6.7	26.8	7.9	0.000
***Cardiovascular risk factors***					
Daily cigarette smoking	20.0		3.1		0.000
Alcohol on ≥6 days/month	19.5		5.7		0.000
Physical activity >3000 MET-min/wk	64.4		18.4		0.000
Systolic blood pressure (mmHg)	121.2	11.2	110.5	10.0	0.000
Diastolic blood pressure (mmHg)	67.0	8.1	69.1	8.0	0.008
Total cholesterol (mmol/l)	4.06	0.81	4.23	0.86	0.031
LDL-cholesterol (mmol/l)	2.28	0.74	2.56	0.81	0.000
HDL-cholesterol (mmol/l)	1.46	0.43	1.36	0.39	0.011
Triglycerides (mmol/l)	0.69	0.28	0.69	0.28	ns
Glucose (mmol/l)	5.24	0.58	4.96	0.58	0.000
Uric acid (mmol/l)	0.33	0.08	0.24	0.70	0.000

P values are indicated in italic.

BMI, waist and WHtR correlated highly with each other (correlation coefficient ~0.9, p< 0.001) in both males and females. These three indices also correlated highly with body fat mass in females (0.84-0.90) but less strongly in males (~0.7). The correlation coefficients with WHiR were markedly lower (<0.5), but still highly significant (p<0.001).

The prevalence of CVRFs increased in a dose response manner across increasing categories of all obesity indices except for HDL-cholesterol (males and females) and blood glucose (males) (Table 2). Associations were weakest for WHiR. For most of the associations, the prevalence increased by an order of 50%-300% when comparing a CVRF between low (<P50) vs. high (>P90) categories of the obesity indices. The associations with the CVRFs were generally strong with BMI.

**Table 2:** Prevalence of risk factors according to categories of different obesity indices

	**Males**					**Females**				
	**BMI**	**Waist**	**WHiR**	**WHtR**	**Fat%**	**BMI**	**Waist**	**WHiR**	**WHtR**	**Fat%**
Blood pressure ≥120/80 mmHg (M: 52.3%; F: 21.9%)*										
P <50	39.7	40.6	49.5	41.2	43.3	10.5	9.1	22.8	14.9	9.6
P 50-79	62.6	58.3	45.7	57.6	59.3	30.4	36.7	22.1	24.6	31.9
P 80-89	75.0	75.0	72.2	75.0	60.0	39.1	25.9	26.0	34.8	40.9
P 90+	63.1	68.4	68.4	68.4	68.4	36.4	34.7	13.0	36.4	34.7
	*0.004*	*0.007*	*ns*	*0.009*	*0.080*	*0.000*	*0.000*	*ns*	*0.037*	*0.000*
LDL-cholesterol ≥3.0 mmol/l (M: 10.8%; F: 29.0%)										
P <50	3.1	5.2	6.1	6.2	9.3	23.7	27.3	28.1	25.4	23,7
P 50-79	15.5	11.7	15.2	8.5	10.2	27.5	26.4	26.4	27.5	27.5
P 80-89	15.0	20.0	11.1	25.0	15.0	56.5	33.3	34.7	43.5	45.4
P 90+	31.6	26.3	21.1	26.3	15.8	31.8	39.1	34.7	36.4	43.5
	*0.004*	*0.021*	*ns*	*0.010*	*ns*	*0.017*	*ns*	*ns*	*ns*	*0.076*
HDL-cholesterol <1.03 mmol/l (M: 14.4%; F: 19.3%)										
P <50	8.1	11.4	17.2	10.3	8.2	16.7	13.6	14.9	13.2	18.4
P 50-79	20.7	15.0	11.9	16.9	20.3	23.2	25.0	20.5	29.0	20.2
P 80-89	10.0	15.0	11.0	10.0	20.0	30.4	30.9	30.4	26.1	27.3
P 90+	31.6	26.3	10.5	31.6	21.0	10.0	17.3	26.1	13.6	13.0
	*ns*	*ns*	*ns*	*ns*	*ns*	*ns*	*ns*	*0.018*	*ns*	*Ns*
Triglycerides ≥1.00 mmol/l (M: 10.3%; F: 12.7%)										
P <50	5.1	6.5	6.1	7.2	7.2	5.2	4.5	12.3	4.3	7.0
P 50-79	8.6	6.7	8.5	3.4	5.1	15.9	16.1	10.3	15.9	10.1
P 80-89	20.0	25.0	16.7	20.0	20.0	21.7	18.5	21.7	21.7	27.3
P 90+	31.6	26.3	31.6	36.8	31.6	31.8	34.8	13.0	36.4	34.8
	*0.002*	*0.006*	*0.006*	*0.000*	*0.003*	*0.002*	*0.000*	*ns*	*0.000*	*0.000*
Glycemia ≥5.6 mmol/l (M: 23.1%; F: 8.8%)										
P <50	24.5	26.0	23.2	24.7	21.6	5.3	5.4	7.0	6.1	3.5
P 50-79	24.1	20.0	22.0	20.3	28.8	4.3	5.8	10.3	4.3	7.2
P 80-89	10.0	15.0	27.8	30.0	25.0	17.3	14.8	13.0	13.0	27.3
P 90+	26.3	26.3	21.0	15.7	10.5	31.8	26.0	8.8	31.8	21.7
	*ns*	*ns*	*ns*	*ns*	*ns*	*0.000*	*0.007*	*ns*	*0.000*	*0.000*
Uric acid >0.39 (M) or > 0.31 (F) μg/l (M: 20%; F: 20%)										
P <50	14.3	11.4	14.1	13.4	13.4	14.9	15.5	20.2	16.7	15.8
P 50-79	15.5	21.7	30.5	20.3	20.3	23.2	20.5	16.2	18.8	17.4
P 80-89	20.0	25.0	5.5	20	25.0	17.3	22.2	30.4	17.3	31.8
P 90+	52.6	42.0	21.0	42.1	36.8	40.9	39.1	21.7	45.4	39.1
	*0.001*	*0.012*	*ns*	*0.034*	*ns*	*0.04*	*ns*	*ns*	*0.02*	*0.034*

* The overall prevalence of cardiovascular risk factors (CVRFs) is displayed between parentheses for males (M) and females (F).

BMI: body mass index; WHiR; waist/hip ratio; WHtR: waist/height ratio; Fat%: percent body fat (bioimpedance).

P values are indicated in italic.

Based on continuous variables (linear regression), all obesity indices except WHiR were associated with all CVRFs, except for LDL-cholesterol in females and blood glucose in males (Table 3). The regression coefficients were high for BMI in most instances, although the associations with BMI were not markedly different than the associations with waist, WHtR (particularly in males) and body fat mass for several CVRFs. The standardized regression coefficients (range from 0 to 1) relating BMI and CVRFs were fairly high, e.g. 0.25-0.36 for BP, 0.26-0.40 for triglycerides, 0.37 for blood glucose (females), and 0.27-0.34 for uric acid. Associations were weaker for HDL-cholesterol in both sexes (e.g. 0.17-0.21 for BMI). The associations between the obesity indices and CVRFs were virtually unchanged when adjusting for smoking, alcohol intake, and physical activity (results not shown).

**Table 3:** Standardized linear regression coefficients for the association between obesity indices and cardiovascular risk factors

	**Males**					**Females**				
	**BMI**	**Waist**	**WHiR**	**WHtR**	**Fat%**	**BMI**	**Waist**	**WHiR**	**WHtR**	**Fat%**
Systolic BP	0.25***	0.25***	0.09ns	0.23**	0.19**	0.30***	0.29***	0.12ns	0.23***	0.34***
Diastolic BP	0.25***	0.24***	0.10ns	0.25**	0.15*	0.36***	0.32***	0.10ns	0.29***	0.40***
LDL-cholesterol	0.24**	0.19*	0.16*	0.23**	0.17*	0.06ns	0.04ns	0.00ns	0.08ns	0.12*
HDL-cholesterol	−0.21**	−0.12ns	0.00ns	−0.15*	−0.16*	−0.17**	−0.19**	−0.17*	−0.22**	−0.14*
Triglycerides	0.40***	0.37***	0.20**	0.41***	0.29***	0.26***	0.29***	0.08ns	0.31***	0.26***
Fasting glucose	0.05ns	0.03Ns	0.06ns	0.07ns	0.06ns	0.37***	0.36***	0.21**	0.36***	0.25***
Uric acid	0.34***	0.35***	0.23**	0.37***	0.27***	0.27***	0.30***	0.15*	0.30***	0.25***

BMI: body mass index; WHiR; waist/hip ratio; WHtR: waist/height ratio; Fat%: fat percent (bioimpedance).

Each line represents a different model, separately in males and in females.

* p<0.05; ** p <0.01; *** p <0.001.

In ROC analysis (Table 4), the AUC ranged consistently between 60% and 70% for the probability that the indices of obesity correctly identified subjects with CVRFs, except for individuals with low HDL-cholesterol (females) and high blood glucose (males). The highest AUC was observed for triglycerides (e.g. 75% for BMI among males and consistently >70% for several other indices had elevated triglycerides). Overall, BMI performed well in predicting individuals with CVRFs.

**Table 4:** AUC values for obesity indices for the prediction of elevated cardiovascular risk factors

	**Males**					**Females**					**Indices with highest AUC**	
	**BMI**	**Waist**	**WHiR**	**WHtR**	**Fat%**	**BMI**	**Waist**	**WHiR**	**WHtR**	**Fat%**	**Males**	**Females**
BP ≥120/90	66.1	67.2	58.1	64.7	61.3	69.8	69.4	53.8	66.1	69.7	W, BMI, WHtR	BMI, F%, W
	58-73	60-75	50-66	57-72	53-69	62-77	62-77	46-62	58-74	62-77		
LDL-cholesterol ≥3.0	72.5	70.9	67.1	68.5	62.9	58.9	56.1	52.9	56.9	58.6	BMI, W	BMI, F%
	61-84	60-82	55-79	56-81	50-75	51-67	48-64	44-61	49-65	50-67		
HDL-cholesterol <1.0	62.6	54.9	48.1	58.7	62.5	55.5	56.9	56.7	59.5	50.3	BMI, F%	WHtR, WHiR
	51-73	43-61	41-58	45-63	52-73	47-63	48-64	50-67	51-68	43-59		
Triglycerides ≥1.0	74.6	70.1	72.0	69.7	67.8	71.1	73.2	57.4	73.2	70.2	BMI, WHiR, W	WHt, W, BMI
	64-86	57-83	60-83	55-84	54-82	60-82	64-83	47-67	63-83	59-81		
Glycemia ≥ 5.6	48.9	45.8	50.9	50.9	50.0	72.5	71.2	58.6	69.8	74.3	-	F%, BMI, W
	39-59	36-55	42-60	41-60	41-59	61-85	58-84	46-72	57-83	63-85		
Uric acid ≥P80	63.9	65.7	57.3	64.8	63.0	58.6	60.1	53.8	61.5	59.9	W, WHtR, BMI	WHtR, W, F%
	53-74	56-76	48-67	55-74	52-73	48-68	50-70	44-63	52-71	50-70		

AUC: area under the receiver operator characteristics (ROC) curve.

BMI: body mass index; WHiR: waist/ratio; WHtR: waist/height ratio; Fat%: fat percent.

The mean number of CVRFs (range: 0–6) and the prevalence of combinations of CVRFs according to categories of the different obesity indices is shown in Table 5. The proportion of youth with several CVRFs increased markedly along increasing categories of obesity for all obesity indices. Of note, the displayed values correspond to the predictive positive value for the test set at the mentioned BMI levels (e.g. there is a 73.7% probability that a male with a BMI ≥P90 has ≥2 CVRFs).

**Table 5:** Mean score of cardiovascular risk factors (CVRF) and proportions with ≥2 CVRFs and ≥3 CVRFs according to categories of obesity indices

	**Males**					**Females**				
	**BMI**	**Waist**	**WHiR**	**WHtR**	**Fat%**	**BMI**	**Waist**	**WHiR**	**WHtR**	**Fat%**
Mean score										
P <50	1.13	1.15	1.31	1.40	1.19	0.67	0.64	0.91	0.68	0.68
P 50-79	1.59	1.53	1.46	1.49	1.68	1.06	1.14	0.96	1.07	1.01
P 80-89	1.75	1.95	1.78	2.10	1.70	1.73	1.33	1.26	1.43	1.73
P 90+	2.47	2.26	1.95	2.32	1.95	1.50	1.60	1.00	1.68	1.61
	*0.000*	*0.000*	*0.000*	*0.000*	*0.000*	*0.000*	*0.000*	*0.003*	*0.000*	*0.000*
Score ≥ 2CVRFs (M: 44.1%, F: 28.1%)										
P <50	35.7	35.4	39.4	34.0	36.1	15.8	15.4	27.2	16.7	1.7
P 50-79	46.5	46.7	45.8	45.7	54.2	31.9	36.8	30.9	31.9	31.9
P 80-89	50.0	55.0	55.6	65.0	45.0	60.9	44.4	34.7	52.2	54.5
P 90+	73.7	68.4	52.6	68.4	52.6	45.5	43.5	17.4	50.0	47.8
	*0.019*	*0.035*	*ns*	*0.007*	*ns*	*0.000*	*0.001*	*ns*	*0.000*	*0.000*
Score ≥ 3CVRFs (M: 14.9%, F: 7.0%)										
P <50	6.1	8.3	11.1	7.2	6.1	0.0	0.0	7.9	0.0	0.0
P 50-79	19.0	13.3	13.5	11.9	22.0	8.9	8.8	4.4	8.7	8.7
P 80-89	20.0	30.0	27.8	45.0	25.0	17.3	14.8	8.7	13.0	18.0
P 90+	42.1	36.8	26.3	31.6	26.3	27.3	26.1	8.7	31.8	26.0
	*0.000*	*0.003*	*ns*	*0.000*	*0.008*	*0.000*	*0.000*	*ns*	*0.000*	*0.001*

Score ranges between 0–6 according to the presence of 6 CVRFs: BP ≥120/80 mmHg; LDL-cholesterol ≥3 mmol/l; HDL <1.03 mmol/l; triglycerides ≥1.0 mmol/l; glucose ≥5.6 mmol/l; uric acid > sex-specific percentile 80.

P values are indicated in italic.

We calculated that to predict the presence of ≥2CVRFs among male/female youth, a BMI cutoff set at ≥P80 had a sensitivity of 28%/37%, a specificity of 86%/87%, a positive predictive value of 62%/53% and a negative predictive value of 60%/78%. The positive predictive value of such a test to detect ≥2 CVRFs (62%/53%) was only slightly larger than the *a priori* chance to classify individuals with ≥2 CVRFs in the population, which equates to the overall prevalence of individuals in the population with ≥2 CVRFs, i.e. 44% of males and 28% of females (Table 5). The sensitivity of this test is low because the large majority of individuals with ≥2 CVRFs are found among the 80% of persons with BMI <P80.

## Discussion

We found a high prevalence of several CVRFs, including obesity, in late adolescents in the Seychelles, with marked differences between males and females. All the obesity markers studied were strongly associated with most of the CVRFs, except for WHiR. BMI performed at least as well as the other indices for the prediction of single or combined CVRFs, with a few exceptions.

The high prevalence of CVRFs, including obesity, in youths in Seychelles is consistent with findings from other surveys in children [20] and in adults [25] in Seychelles. A high prevalence of several CVRFs, particularly obesity, is increasingly well documented in adults in Africa [26]. This study contributes new information on the prevalence of CVRFs in youth, an age category where limited data are available in the region, particularly with regards to biological markers such as blood lipids and uric acid. Our finding of a high prevalence of CVRFs in youths reiterates the importance of a life course approach for prevention and control of cardiovascular disease [26].

The distribution of several CVRFs differed markedly according to sex in this study. This finding may relate to differences in body composition, e.g. higher fat mass among females and higher muscular mass among males. We found higher systolic BP and higher blood glucose in males than females, but similar levels of triglycerides. We also found lower LDL-cholesterol in males than in females which is consistent with studies of youth in other countries [27]. The sex difference in systolic BP may relate to the large height difference between males and females, since height is an important driver of BP in youth [28]. Although recent guidelines consider same cutoffs for males and females at age 18–21 [24], sex-specific cutoffs could be more appropriate at the transition between adolescence and adulthood in view of the twofold higher prevalence of elevated BP in male than female young adults in our study, and this despite higher obesity in females than in males. The higher levels of glucose in males than in females, despite higher levels of obesity (a marker for increased insulin resistance) in males could be partly related to a poorer fasting state among males. It is also compatible with fasting blood glucose being a poor marker of insulin resistance [29]. Higher levels of HDL-cholesterol in males than in females in our study is consistent with results reported in adults in Seychelles [25], but it contrasts with higher levels of HDL-cholesterol in females than in males in several other populations [30]. Serum triglycerides level was low in young adults of Seychelles which is consistent with a trend toward lower triglycerides values in blacks than in whites [31]. It is not yet clear whether uric acid is an independent CVRF, but strong evidence supports a causal role of uric acid in the development of hypertension. This association is particularly strong in younger individuals [32]. Serum uric acid concentration is higher in males than in females in all populations, which may relate to higher muscular metabolic turnover. These substantial sex differences in the distribution of several CVRFs among late adolescents emphasize the importance of conducting epidemiological studies in different populations among all age groups.

All indices of obesity, except WHiR, were strongly associated with most of the considered CVRFs. These findings concur with studies in youth in Western countries [6,14]. Lower performance with WHiR can relate to the larger likelihood for random variation and/or bias in the measurements of both waist and hip, and perhaps particularly for hip (as we found in our data), compared to easier and more standardized measurements of height, weight and the related indices. These associations were of similar magnitude whether the obesity indices were intended to assess central fat mass (waist, WHtR) or overall fat mass (BMI, percent fat) and whether the CVRFs were essentially vascular (BP) or metabolic (blood lipids, glycemia). Overall, BMI performed as well as the other obesity indices for the prediction of CVRFs. The findings are robust since we found similar findings whether the analyses were based on categorical variables (which do not assume a dose response relationship) or continuous variables (which assume a dose response relationship). The good performance of BMI compared to other obesity indices like waist circumference or WHtR is not surprising in view of the high correlation between the indices we studied. Bouchard noted that all simple obesity indices (e.g. BMI, fat mass, and waist) provide essentially similar information because they correlate equally well both with each other (r >0.90) and with CT-scan measured abdominal visceral fat, but less well (~0.7-0.8). Most of the variance of obesity-related anthropometrics is captured by BMI, and all these indices are similarly strong surrogates of fat mass [33]. Furthermore, total fat mass and muscular mass (perhaps better captured by BMI) can be just as important as visceral fat mass (perhaps better captured by waist circumference) when predicting the presence of concomitant CVRFs. Of note, sex differences in the ability of the obesity indices to predict some CVRFs emphasize the need to analyze associations between obesity indices and CVRFs, and their related discriminating power to predict CVRFs, by sex in this young age range.

Despite fairly strong associations, the obesity indices had limited ability to predict CVRFs. For example, a male youth had a 74% chance to have ≥2 CVRFs if he was overweight (BMI >P90) but as much as 36% if he was lean (BMI <P50) (Table 5). This translated into a poor sensitivity: a test using BMI above percentile 80 would miss most individuals with ≥2 CVRFs in the population. These findings are consistent with the fact that any measured characteristic must be very tightly associated with an outcome to be a good screening tool [34]. The US Preventive Services Task Force has recommended that children and adolescents are screened for obesity using BMI. This follows from the strong association of BMI with body fat and the availability of effective interventions for weight control at the individual level in youth [35]. The potential use of obesity indices to screen for the presence of CVRFs in youths is, however, a distinct issue. Because they are simple, reliable and inexpensive metrics, one should evaluate whether obesity indices, and particularly BMI, could be used as filters in stepwise strategies for the identification of CVRFs [36]. This might be particularly helpful in low resources settings.

Strengths of the study include the population-based design, the fairly large sample size considering its reliance on blood samples in healthy young individuals, and the availability of a broad panel of CVRFs. The study also has some limitations. We cannot be sure that participants were fasting, which may weaken associations with biological markers. We took only one measurement of the anthropometric indices, and we did not assess differences in measurements between the observers during the study. However, all our observers had long experience with epidemiological research. Furthermore, the goal of the study was to examine the performance of adiposity indices to predict the presence of concomitant CVRFs: it is likely that these conditions (one single measurement for the adiposity indices and good but perhaps not optimal skills of the observers for these measurements) correspond to a situation commonly found in clinical practice. The sample size was not adequate for precise analysis among extreme categories of overweight [6]. Of note, the cross-sectional design is not a limitation when predicting CVRFs from current indices of obesity.

## Conclusion

In conclusion, we found an increased prevalence of obesity and CVRFs in late adolescence in the Seychelles. We also found that BMI performed generally at least as well as the other obesity indices to predict the presence of single or combined CVRFs at this young age in this population. These findings highlight the broad metabolic derangements of obesity in youth and the subsequent need for appropriate weight control interventions at the individual and population levels. Further epidemiological studies should evaluate whether obesity indices could be combined with other markers to provide efficient screening tests of cardiovascular risk in youths and young adults.

## Abbreviations

CVRF: Cardiovascular Risk Factor; BMI: Body Mass Index; WHiR: Waist/Hip Ratio; WHtR: Waist/Height Ratio; BP: Blood Pressure; LDL-cholesterol: Low Density Lipoprotein-Cholesterol; HDL-cholesterol: High Density Lipoprotein-Cholesterol; ROC: Roc Operating Characteristics; AUC: Area Under the Curve.

## Competing interests

The authors declare that they have no competing interests.

## Authors’ contributions

PB participated in the study design, study analysis and drafting of the manuscript. TA participated in the study analysis and drafting of the manuscript. BV participated in the conduct of the study and revision of the manuscript. GM participated in the study design, study funding and revision of the manuscript. All authors read and approved the final manuscript.

## Pre-publication history

The pre-publication history for this paper can be accessed here:

http://www.biomedcentral.com/1471-2431/12/176/prepub
